# Characteristics of non-accidental injuries in children and adolescents in Asia: a cross-national, multicenter cohort study

**DOI:** 10.1038/s41598-023-33471-x

**Published:** 2023-04-23

**Authors:** Po-Yuan Wang, Wei-Chieh Tseng, Meng-Chang Lee, Li-Min Hsu, Sang Do Shin, Sabariah Faizah Jamaluddin, Hideharu Tanaka, Do Ngoc Son, Ki Jeong Hong, Sattha Riyapan, Ali Haedar, Wen-Chu Chiang, The PATOS Clinical Research Network, Ramana Rao, George P. Abraham, T. V. Ramakrishnan, Sabariah Faiwah Jamaluddin, Mohd Amin Bin Mohidin, Al-Hilmi Saim, Lim Chee Kean, Cecilia Anthonysamy, Shah Jahan Din Mohd Yssof, Kang Wen Ji, Cheah Phee Kheng, Shamila bt Mohamad Ali, Periyanayaki Ramanathan, Chia Boon Yang, Hon Woei Chia, Hafidahwati binti Hamad, Samsu Ambia Ismail, Wan Rasydan B. Wan Abdullah, Hideharu Tanaka, Akio Kimura, Bernadett Velasco, Carlos D. Gundran, Pauline Convocar, Nerissa G.Sabarre, Patrick Joseph Tiglao, Ki Jeong Hong, Kyoung Jun Song, Joo Jeong, Sung Woo Moon, Joo-yeong Kim, Won Chul Cha, Seung Chul Lee, Jae Yun Ahn, Kang Hyeon Lee, Seok Ran Yeom, Hyeon Ho Ryu, Su Jin Kim, Sang Chul Kim, Li-Min Hsu, Jen Tang Sun, Ruei-Fang Wang, Shang-Lin Hsieh, Wei-Fong Kao, Sattha Riyapan, Parinya Tianwibool, Phudit Buaprasert, Osaree Akaraborworn, Omer Ahmed Al Sakaf, Saleh Fares LLC, Le Bao Huy, Do Ngoc Son, Nguyen Van Dai, T. V. Ramakrishnan, Sabariah Faiwah Jamaluddin, Hideharu Tanaka, Bernadett Velasco, Ki Jeong Hong, Jen Tang Sun, Pairoj Khruekarnchana, Saleh Fares LLC, Do Ngoc Son

**Affiliations:** 1 Department of Pediatrics, Far Eastern Memorial Hospital, New Taipei City, Taiwan; 2 Graduate Institute of Clinical Medicine, Medical College, National Taiwan University, Taipei, Taiwan; 3 Department of Emergency Medicine, National Taiwan University Hospital, No. 7, Chung-Shan South Road, Taipei, 100 Taiwan; 4 Department of Traumatology and Critical Care, National Taiwan University Hospital, Taipei, Taiwan; 5 Department of Emergency Medicine, Seoul National University College of Medicine and Hospital, Seoul, Korea; 6 Faculty of Medicine, Universiti Teknologi MARA, Batu Caves, Malaysia; 7 Graduate School of Emergency Medical Service System, Kokushikan University, Tokyo, Japan; 8 Center for Critical Care Medicine, Bach Mai Hospital, Hanoi, Vietnam; 9 Department of Emergency and Critical Care Medicine, Hanoi Medical University, Hanoi, Vietnam; 10 Faculty of Medicine, University of Medicine and Pharmacy, Vietnam National University, Hanoi, Vietnam; 11 Department of Emergency Medicine, Faculty of Medicine, Siriraj Hospital, Bangkok, Thailand; 12 Department of Emergency Medicine, Faculty of Medicine, Universitas Brawijaya, Malang, Indonesia; 13 Department of Emergency Medicine, National Taiwan University Hospital Yunlin Branch, Douliu City, Taiwan; 14 GVK EMRI, Hyderabad, India; 15 Indian Institute of Emergency Medical Services, Chennai, India; 16 Sri Ramachandra Medical Center, Chennai, India; 17 Sungai Buloh Hospital, Sungai Buloh, Malaysia; 18 Sultanah Aminah Hospital, Johor, Malaysia; 19 Seri Manjung Hospital, Seri Manjung, Malaysia; 20 Pulau Pinang Hospital, Pulau Pinang, Malaysia; 21 Serdang Hospital, Kajang, Malaysia; 22 Kuala Lumpur Hospital, Kuala Lumpur, Malaysia; 23 Sarikei Hospital, Sarikei, Malaysia; 24 Sabah Women and Children’s Hospital, Kota Kinabalu, Malaysia; 25 Ampang Hospital, Ampang, Malaysia; 26 Kajang Hospital, Kajang, Malaysia; 27 Miri Hospital, Miri, Malaysia; 28 Sarawak General Hospital, Kuching, Malaysia; 29 Queen Elizabeth II Hospital, Kota Kinabalu, Malaysia; 30 Teluk Intan Hospital, Teluk Intan, Malaysia; 31 Raja Perempuan Zainab II Hospital, Kota Bharu, Malaysia; 32 National Center for Global Health and Medicine Hospital, Tokyo, Japan; 33 East Avenue Medical Center, Quezon City, Philippines; 34 Philippine College of Emergency Medicine, Parañaque, Philippines; 35 Southern Philippines Medical Center, Davao, Philippines; 36 Pasig City General Hospital, Pasig, Philippines; 37 Corazon Locsin Montelibano Memorial Regional Hospital, Bacolod, Philippines; 38 Boramae Medical Center, Seoul, South Korea; 39 Seoul National University Bundang Hospital, Seoul, South Korea; 40 Korea University Ansan Hospital, Ansan, South Korea; 41 Samsung Medical Center, Seoul, South Korea; 42 Dongguk University Ilsan Hospital, Goyang, South Korea; 43 Kyungpook National University Hospital, Daegu, South Korea; 44 Wonju Severance Christian Hospital, Wonju, South Korea; 45 Pusan National University Hospital, Busan, South Korea; 46 Chonnam National University Hospital, Gwangju, South Korea; 47 Korea University Anam Hospital, Seoul, South Korea; 48 Chungbuk National University Hospital, Cheongju, South Korea; 49 Department of Emergency Medicine, Far Eastern Memorial Hospital, New Taipei City, Taiwan; 50 Shin Kong Wu Ho-Su Memorial Hospital, Taipei, Taiwan; 51 Mackay Memorial Hospital, Taipei, Taiwan; 52 Taipei City Hospital, Taipei, Taiwan; 53 Faculty of Medicine, Chiangmai University, Chiangmai, Thailand; 54 Faculty of medicine Vajira Hospital, Navamindradhiraj University, Bangkok, Thailand; 55 Prince of Songkla University, Hat Yai, Thailand; 56 Dubai Corporation for Ambulance Services, Dubai, United Arab Emirates; 57 National Ambulance, Abu Dhabi, United Arab Emirates; 58 Thong Nhat Hospital, Ho Chi Minh City, Vietnam; 59 Viet Tiep Hospital, Haiphong, Vietnam; 60 Rajavithi Hospital, Bangkok, Thailand

**Keywords:** Epidemiology, Paediatric research

## Abstract

Children and adolescents are vulnerable to non-accidental injury. Early identification and prevention rely on detailed epidemiological studies, which are limited in Asia. This retrospective study used the registry data of Pan-Asian Trauma Outcome Study (PATOS) from October 1, 2015 to December, 31, 2020. Pediatric patients (aged < 20 years) with non-accidental injuries were enrolled, which were divided by age into preschool (0–6 years), child (7–12 years), and adolescent (13–19 years) groups. Baseline characteristics, injury epidemiology, and excess mortality ratio-adjusted injury severity score (EMR-ISS) were collected. Major trauma was defined as an EMR-ISS score > 24. The study enrolled 451 patients with non-accidental injuries, accounting for 2.81% of pediatric trauma events presented to an emergency department in the PATOS registry. The overall mortality rate was 0.9%, similar to those in Western countries. Mortality rate was high in preschool children (8.7%, *p* = 0.017) than in other age groups. The sex-specific incidence was higher in boys (3.10% vs. 2.13%, *p* = 0.001). In adolescents, more events occurred on the street (25.9%), whereas home remained the most common locale in girls of all ages. In the multivariable regression analysis, abdominal and multiple injuries were risk factors for major trauma.

## Introduction

Non-accidental injury events are an important public health concern. These events cause significant physical injury, medical resource consumption, and future loss of productivity^1–3^. Children and adolescents are especially vulnerable to non-accidental events^4,5^.

The epidemiology of non-accidental pediatric injuries is influenced by age and sex. In previous studies, non-accidental injuries accounted for 1.1 to 20% of all injuries and caused significant morbidity and mortality in the pediatric population^2,4,6,7^. The majority of patients in the pediatric population were adolescents, followed by infants^5^. The mortality rate of non-accidental injuries ranges from 0.1 to 8% and is associated with risk factors such as sex, age, and ethnicity^3–5,8^. Those who are injured in non-accidental injury events often seek medical help in the emergency department (ED)^9,10^. Early identification of the victims would help in the timely involvement of the social protection system, facilitate the judicial system to maintain justice, and prevent further damages caused by the offenders. Furthermore, understanding the demographics of non-accidental injuries in a vulnerable populations would help develop preventive strategies, reduce the incidence of injury, and plan resource allocation^3,9^.

Therefore, the characteristics of non-accidental injuries in children and adolescents are of great importance. However, the epidemiological profiles of injury patterns and outcomes of non-accidental injuries are limited^10,11^. Data of the Asian pediatric population remain unclear, especially in the ED setting^3,11^. In addition, firearm injuries is a severe threat in the pediatric population in the United States^12,13^. The epidemiology of firearm injuries in Asia, where the firearm control policy is stricter than that in the United States, remains unclear. Thus, the aim of our study was to delineate the incidence, patient characteristics, and severity of non-accidental injuries in children and adolescents presented to the ED in Asia.

## Methods

### Design and settings

This retrospective study using registered data was conducted at the participating centers of the Pan-Asian Trauma Outcome Study (PATOS). The study period was from October 1, 2015, to December 31, 2020, registering 127,715 injury events. Phase I data enrolled 71,262 injury events from October 2015 to November 2018, and phase II data enrolled 56,453 events from December 2018 to December 2020, respectively. The PATOS is a clinical research network with a multicenter trauma registry in Asia^14,15^, which records patient data, including baseline characteristics, injury epidemiology, prehospital care, emergency department care, hospital care, injury severity, and outcomes. Data were recorded in an unified manner. The registry used a standardized electronic data form with consensus variables. The study protocol we devised adhered to ethical guidelines of the 1975 Declaration of Helsinki, securing a priori approval of Research Ethics Committee Office of National Taiwan University Hospital, which approved the study protocol and waived the need for written informed consent. All the other centers in the PATOS collaboration independently obtained ethical approval.

### Participants

In the screening process, only participants younger than 20 years at the time of injury and had “assault” as a recorded intention of the injury or “sexual assault” as a recorded mechanism of injury were eligible for inclusion. Participants with injuries not due to non-accidental injury or unknown age were excluded.

### Variables

The baseline characteristics of the participants, including age, sex, and country, were collected. Data on injury epidemiology were collected, including mechanism of injury, type of injury, location of the body in the injury, and place where the injury occurred. Data on alcohol and psychoactive drug/substance use in the events of patients and injurers were also collected.

To better understand the effect of age on the epidemiology of non-accidental injuries, we divided the patients into three groups: preschool children (0–6 years), children (7–12 years), and adolescents (13–19 years). Injury severity was categorized on the basis of the excess mortality ratio-adjusted injury severity score (EMR-ISS) as mild (1 ≤ EMR-ISS ≤ 8), moderate (9 ≤ EMR-ISS ≤ 24), and severe and critical (EMR-ISS > 24)^16,17^. For the purpose of analysis of major trauma and its risk factors, events with moderate EMR-ISS were combined with those with mild EMR-ISS as non-major trauma group as previous studies^17–19^. Major trauma was defined as EMR-ISS > 24. The characteristics were further compared, and the risk factors for severe injuries were analyzed.

### Statistical methods

Statistical analyses were performed using SPSS Statistics version 20 for Windows (2011, IBM, Armonk, NY, USA) and R version 4.0.3. In our presentation, all the continuous variables are presented as median (interquartile range, IQR), and the categorical variables are presented as numbers (%), unless otherwise specified. For comparing continuous and categorical variables, the Mann–Whitney *U* test and the chi-square or Fisher exact test was used, respectively. For the risk factor analysis for major trauma, basic characteristics, including age, sex, and variables of injury mechanism and injured body region with p values < 0.05 in the univariable analysis were selected for the multivariable regression analysis. A *p* value less than 0.05 was considered statistically significant.

## Results

**Figure 1:**
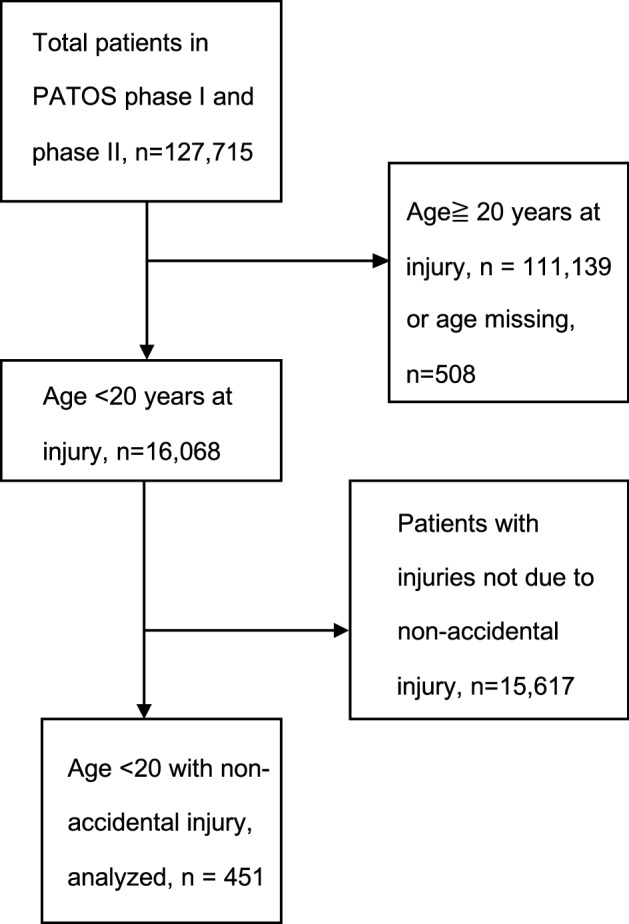
Study algorithm of patients included in the study. *PATOS* Pan-Asian Trauma Outcome Study.

In the Phase I and Phase II PATOS databases, 127,715 injury events were registered. A total of 451 injuries during the study period were related to non-accidental injuries, which were considered for further analysis (Fig. 1). The nationality distribution of the 451 studied patients was as follows: Korea, 258; Vietnam, 127; Malaysia, 40; Taiwan, 15; Japan, 9; Indonesia, 1; and Thailand, 1.

### Incidence and burden of non-accidental injury events

**Figure 2:**
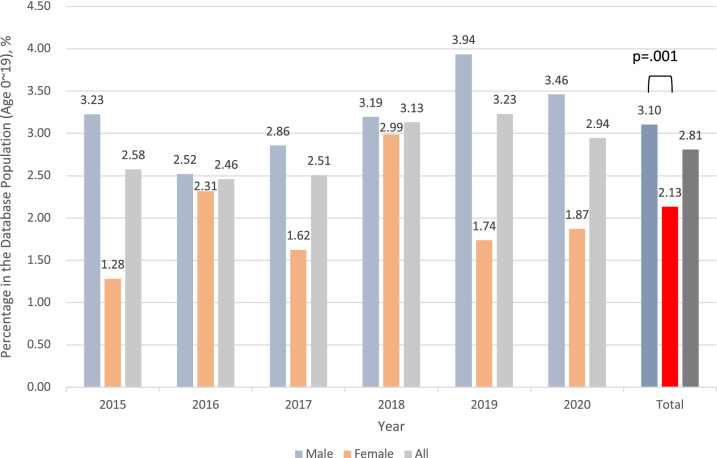
Sex-specific and total percentages of non-accidental injuries among trauma events presenting to emergency departments (age at injury from 0 to 19 years).

Figure 2 shows the percentage of non-accidental injury events among all the trauma events by year. Overall, in the database, 2.81% of trauma events presented to the EDs were non-accidental injury. Male predominance was noted every year and in the total database, and it was more obvious in the adolescent group. However, the differences in the incidence rates between the sexes did not reach statistical significance on analyzing by age group (Supplementary Fig. S1).

### Patient baseline characteristics by age

The baseline characteristics of the three age groups differed (Table 1). The majority of the patient population comprised adolescents (401, 88.9%). The male-to-female ratio was comparable among the three groups. In the adolescent group, more patients suffered from moderate injuries, as categorized according to EMR-ISS, than in the other two groups, whereas in the preschool group, a higher percentage of severe injuries was noted. The places where the non-accidental injury events occurred also significantly differed among the age groups. Most (69.6%) non-accidental injury events that occurred among the preschool patients occurred at home. However, the percentage of injuries at home decreased in the children and adolescent groups, and events occurring on the street and commercial or recreational institutions increased with age (Supplementary Fig. S2). In adolescents, non-accidental injuries most commonly occur on the streets. Sex differences were also observed in the places of injuries. In the male population, the school and street were the most frequent places where non-accidental injury events occurred in children and adolescents, and the home was the most common place of injury among female patients of all age groups (Supplementary Fig. S2). The mechanisms of injury were heterogeneous, particularly in the adolescent group (Table 1). The struck/hit mechanism was the most prevalent mechanism among all the age groups. Stab/cut injuries was the second most common mechanism in adolescents. Only one gunshot event was reported in the study population. In patients, adolescents had more alcohol use in the events (27.2%), but not so with psychoactive drug/substance use (1.7%). In injurers, because the recording was optional, most of them had no information in the database (Supplementary Table S1). Mortality was highest in preschool children (8.7%), whereas no deaths occurred in children, and two died (0.5%) in the adolescent group.

**Table 1:** Baseline characteristics of different age groups.

	Preschool (0 ~ 6 years)	Children (7 ~ 12 years)	Adolescents (13 ~ 19 years)	*p* value
Numbers	23	27	401	–
Male, n (%)	14 (60.9)	21 (77.8)	312 (77.8)	0.067
EMR-ISS, n (%)				**0.014**
1 ~ 8	6 (26.1)	10 (37)	84 (20.9)	0.135
9 ~ 24	9 (39.1)	13 (48.1)	272 (67.8)	**0.003**
> 24	5 (21.7)	3 (11.1)	33 (8.2)	0.074
Missing	3 (13)	1 (3.7)	12 (3)	0.044
Places of injury, n (%)				** < 0.001**
Home	16 (69.6)	9 (33.3)	95 (23.7)	** < 0.001**
School	1 (4.3)	8 (29.6)	36 (9)	**0.005**
Street	3 (13)	6 (22.2)	104 (25.9)	0.395
Commercial	0 (0)	1 (3.7)	48 (12)	0.097
Others	3 (13)	3 (11.1)	118 (29.4)	**0.034**
Mechanism of injury				**0.020**
Sexual assault	1 (4.3)	0 (0)	12 (3)	0.596
Fall	3 (13)	0 (0)	5 (1.2)	**0.010**
Struck/hit	14 (60.9)	23 (85.2)	295 (73.6)	0.152
Stab/cut	2 (8.7)	1 (3.7)	74 (18.5)	0.090
Gun shot	0 (0)	0 (0)	1 (0.2)	> 0.999
Forced choking/hanging	1 (4.3)	0 (0)	4 (1)	0.257
Poisoning	0 (0)	1 (3.7)	1 (0.2)	0.210
Others	2 (8.7)	2 (7.4)	9 (2.2)	0.056
Death, n (%)	2 (8.7)	0 (0)	2 (0.5)	**0.017**

Alcohol and psychoactive drug/substance use were list in Supplementary Table S1.

*EMR-ISS* excess mortality ratio-adjusted injury severity score.

Significant values are in bold.

### Patient baseline characteristics by injury severity and outcomes of injury

The differences between non-major trauma (EMR-ISS ≤ 24) and major trauma (EMR-ISS > 24) were studied (Table 2). The EMR-ISS was not documented in 16 patients (3.55%) in the study population, therefore, these patients were excluded from the subanalysis. The major trauma group had a higher proportion of preschool children. Although the struck/hit mechanism was the most common in both the groups, it was more common in the non-major trauma group. Stab/cut injuries were observed more frequently in the major trauma group. Alcohol use in patients did not affect the trauma severity. The effect of alcohol use in injurers could not be evaluated due to missing data.

**Table 2:** Comparisons of baseline characteristics of non-major and major trauma (N = 435).

Characteristics	EMR-ISS ≦ 24, n (%)	EMR-ISS > 24, n (%)	*p* value
Number of patients	394	41	–
Patient baseline characteristics			
Age (years), median (IQR)	17 (15–19)	17 (15.5–18)	0.689
Preschool (0 ~ 6)	15 (3.8)	5 (12.2)	**0.031**
Children (7 ~ 12)	23 (5.8)	3 (7.3)	0.726
Adolescents (13 ~ 19)	356 (90.4)	33 (80.5)	0.061
Male	304 (77.2)	32 (78)	0.897
Pre-existing disability			
No or mild	365 (92.6)	37 (90.2)	0.72
Moderate or severe	27 (6.9)	4 (9.8)	
Unknown	2 (0.5)	0 (0)	
Alcohol use			
Suspect/confirmed	100 (25.4)	9 (22)	0.63
No use/unknown	294 (74.6)	32 (78)	
Mechanism of injury			**0.003**
Sexual assault	11 (2.8)	1 (2.4)	> 0.999
Fall	6 (1.5)	2 (4.9)	0.169
Struck/hit	299 (75.9)	20 (48.8)	** < 0.001**
Stab/cut	63 (16)	14 (34.1)	**0.003**
Gun shot	1 (0.3)	0 (0)	> 0.999
Choking/hanging	4 (1)	1 (2.4)	0.392
Poisoning	1 (0.3)	0 (0)	> 0.999
Other	9 (2.3)	3 (7.3)	0.094
Body location of injury			** < 0.001**
Head	128 (32.5)	18 (43.9)	0.194
Face	188 (47.7)	10 (24.4)	**0.007**
Neck	17 (4.3)	3 (7.3)	0.421
Thorax	45 (11.4)	10 (24.4)	**0.033**
Abdomen	20 (5.1)	11 (26.8)	** < 0.001**
Spine	3 (0.8)	2 (4.9)	0.072
Upper extremity	78 (19.8)	12 (29.3)	0.222
Lower extremity	31 (7.9)	6 (14.6)	0.425
Skin	8 (2)	3 (7.3)	0.075
Others	6 (1.5)	2 (4.9)	0.169
Multiple locations (≧2)	99 (25.1)	25 (61)	** < .001**
Patient diagnosis and outcome			
Types of injury			** < 0.001**
Fracture	61 (15.5)	12 (29.3)	**0.043**
Sprain	19 (4.8)	1 (2.4)	0.701
Cut	157 (39.8)	22 (53.7)	0.123
Bruise	178 (45.2)	7 (17.1)	** < 0.001**
Burn	1 (0.3)	0 (0)	> 0.999
Concussion	33 (8.4)	5 (12.2)	0.385
Organ injury	11 (2.8)	11 (26.8)	** < 0.001**
Other or unknown	20 (5.1)	4 (9.8)	0.267
Disposition at ED			** < 0.001**
Discharge	326 (82.7)	12 (29.3)	** < 0.001**
Transfer	7 (1.8)	1 (2.4)	0.550
Hospitalization	53 (13.5)	26 (63.4)	** < .001**
Dead at ED	1 (0.3)	1 (2.4)	0.180
Other/unknown	7 (1.8)	1 (2.4)	0.550
Mortality^a^	2 (0.5)	1 (2.4)	0.258

*ED* emergency department, *EMR-ISS* excess mortality ratio-adjusted injury severity score, *IQR* interquartile range.

^a^One with missing EMR-ISS.

Significant values are in bold.

**Figure 3:**
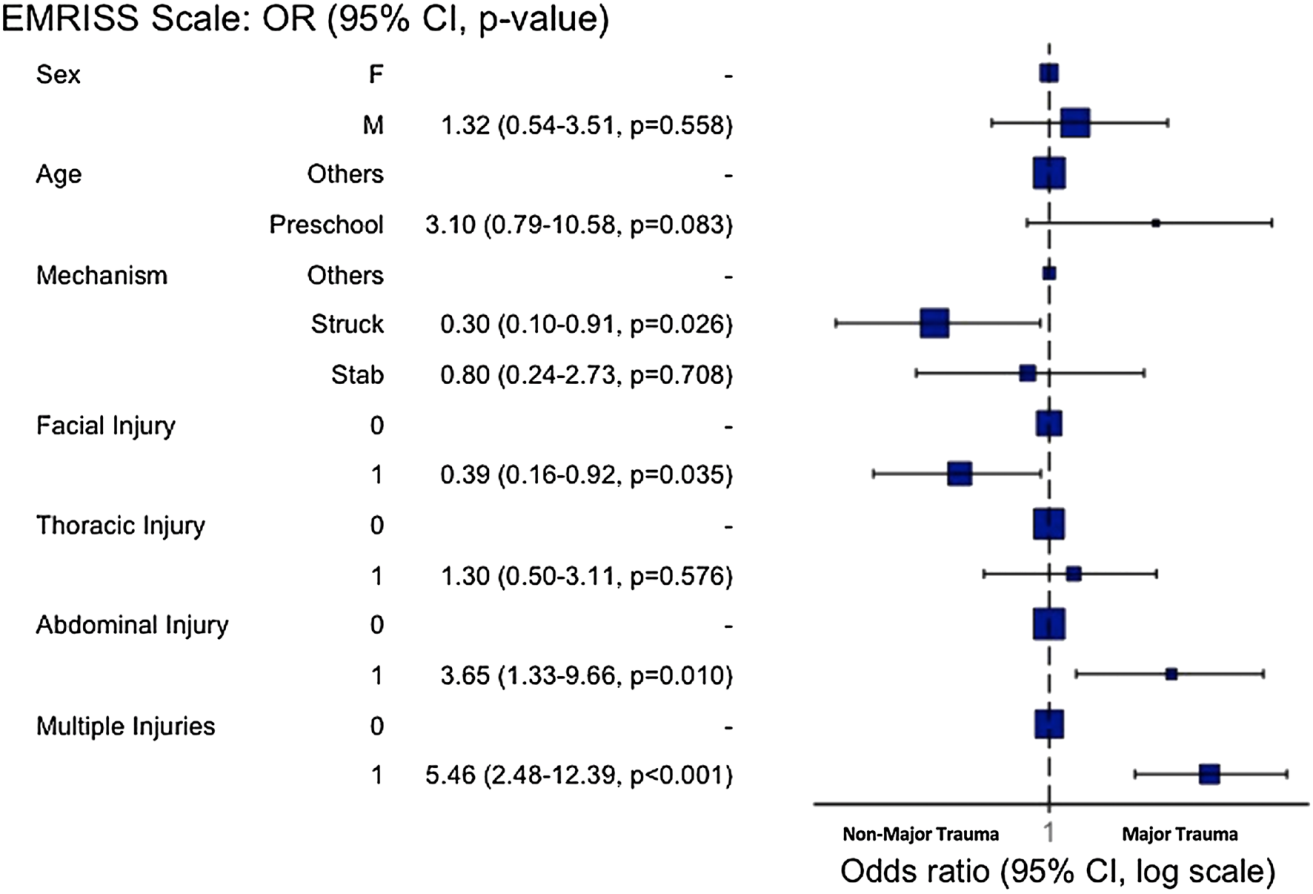
Multivariable regression analysis and adjusted odds ratio of risk factors for non-major trauma versus major trauma. *CI* confidence interval, *EMR-ISS* excess mortality ratio-adjusted injury severity, *OR* odds ratio, 1 present, 0 absent.

Injury outcomes were evaluated (Table 2). The major trauma group had more injuries to the thorax, abdomen, and multiple body parts. Regarding the types of injury, the major trauma group had more fractures and organ injuries. Most of patients with non-major trauma could be discharged from the ED directly (82.7%), but 63.4% of the major trauma cases required hospitalization. In the multivariable regression analysis, the injury mechanism of struck/hit and facial injury served as risk factors for non-major trauma, whereas abdominal and multiple injuries (injury sites ≥ 2) were the risk factors for major trauma (Fig. 3).

Two male infants had ICD-10 codes for intracranial hemorrhage. One was diagnosed of skull fracture, traumatic subarachnoid hemorrhage and epidural hemorrhage, and the mechanism was fall. The other was diagnosed of diffuse traumatic brain injury and traumatic subdural hemorrhage, and the mechanism was struck/hit. Their EMR-ISS were both > 24, accounting for 9% (2 out of 23) of preschool non-accidental injuries and 40% (2 out of 5) of preschool major traumas.

Four patients died due to non-accidental injuries in the study population. The overall mortality rate was 0.9% (4 out of 451). None of the patients had pre-existing comorbidities before the events. Among the four patients, three were male, two were infants, and the rest were adolescents. One infant had a skull fracture, traumatic subarachnoid hemorrhage and epidural hemorrhage due to a fall, and the other had an organ injury in the thorax due to hanging/choking. One adolescent had an organ injury on the head due to a fall, and the other had cuts in the neck and upper arm. Three of the events took place at home, and one occurred in a commercial area. All the patients required cardiopulmonary resuscitation before arriving at the ED, and half of them remained asystole at triage. All the patients received cardiopulmonary resuscitation, and three died in the ED. The details of the four patients were in the Supplementary Table S2.

## Discussion

We report the following important findings: (1) non-accidental injury events accounted for 2.81% of the events among all the injury cases presented to ED in Asian countries; (2) male predominance was noted in the Asian cohort, especially in adolescents; (3) the places where non-accidental injury events took place varied with respect to sex and age; (4) the overall mortality rates for non-accidental injury in children and adolescents were relatively low, but they were high in preschool children; and, (5) abdominal and multiple injuries were risk factors for major trauma. To the best of our knowledge, this is the first report of non-accidental pediatric injuries across Asian countries.

In literature, non-accidental injuries accounted for 1.1 to 20% of all injuries presented to the ED in the Western countries^2,4,6,7^. In the present study, non-accidental injuries accounted for 2.81% of all injuries presenting to the ED, which is consistent with the figures in literature. The incidence during the study period remained consistent, whereas there was a decreasing trend in non-accidental events in the ED in the United States^9,11^. A decrease in pediatric assault injury hospitalizations was also observed in Australia, despite protection orders for children continued to rise^20^. The differences in the incidence trends of non-accidental injury between the United States and Asia might stem from the implementation of evidence-based prevention strategies that reach the youth in the United States. A similar prevention policy may have beneficial effects in Asia.

Our study found that male predominance was significant, especially in adolescents; however, sex was not associated with the risk of developing major trauma or mortality. Previous reports worldwide have also shown a male predominance in non-accidental injuries^1,2,5,20,21^. Higher male-to-female ratio for non-accidental events has been observed in older children and adolescents in the literature^20,22^. In our study, the incidence in male adolescents was higher than that in female adolescents. Therefore, education and interventions to reduce violence are particularly important in boys.

Many studies have shown that the places of injury events differed by age and ethnicity, but sex differences were less discussed^1,3,11,20^. In the present study, preschool children were most likely to be injured at home, but adolescents were more likely to be injured on the streets. This finding is consistent with that of other studies^5,11,23,24^. Furthermore, for the majority of female patients, regardless of age, most non-accidental injury events occurred at home. In contrast, the places of incidence varied more with age for male patients, with the schools and streets being the most common places in male children and adolescents. It was reported in the United States that female accounted for 55.9% when non-accidental events occurred at home, and 29% when on the streets^11^. The variations in the places of incidence with age and sex have not been reported in detail in the literature. Although sex was not associated with major injury outcome, our study provides valuable data on the detailed differences in injury epidemiology among age and sex subgroups, which might be beneficial in the development of injury prevention strategies.

Although preschool children comprised the least proportion of the population in our study, their mortality rate was higher than that of adolescents. Higher mortality or injury severity in young children as compared to other age groups was also reported in previous studies^5,22^ which could be attributed to the vulnerability of young children and delay in the provision of medical care if the caregiver was the assailant^25^. Head trauma is common in pediatric non-accidental trauma^6^, which causes high morbidity and mortality in victims^26,27^. In our data, two infants having major trauma were diagnosed of intracranial hemorrhage, who might be victims of abusive head trauma, including shaken baby syndrome, making up a significant proportion of preschool major traumas (40%) and mortality (25%). Intervention programs dedicated to preventing violence inflicted by caregivers are of paramount importance.

We found that abdominal and multiple injuries were risk factors for major trauma, whereas facial and struck/hit injuries predicted non-major trauma in pediatric non-accidental injuries. In comparison, an Israeli study showed that age less than 1 year and firearm injuries were risk factors for severe violence-related injury, whereas stabbing and unarmed brawling were least likely to cause severe injury^22^. The different variables analyzed in each study partly explained the difference; injured body region was not analyzed in the Israeli study, and ethnicity was not recorded in this study. The epidemiology of firearm injuries may also make a difference. Firearm injuries constituted 5.5% of non-accidental injuries in the Israeli study, but only one case (0.2%) in this pan-Asian study. Firearm injury is a major issue in Western countries, which accounted for 4.6% of all pediatric ED injury visits, and it is the second most common cause of death in children in American trauma centers^5,28,29^. However, this condition was rare in Asia. This difference reflects the influence of the firearm control policy in injury epidemiology. Gunfire was prohibited in most countries participating in the PATOS study. The low percentage of firearm injuries may also have contributed to the lower mortality rate in our cohort. In addition to firearm, alcohol is also a risk factor for non-accidental injury. Alcohol use by victims has been reported to increase interpersonal violence^30^. In our study, alcohol was more frequently used in adolescents than in preschooler and children. However, we had no information on the prevalence of alcohol use in adolescent population; therefore, it was difficult to assess how alcohol influence the incidence of non-accidental injuries. The effect of alcohol use in pediatric non-accidental injury needs more investigation. The epidemiological profile reported by our study is thus valuable in countries where public safety policies are required.

### Limitations

Our study has valuable findings despite certain limitations. First, the definition of non-accidental injury was based on the intentions and mechanisms of the recorded registry data. The detailed judgment and diagnostic processes could not be reviewed. Abusive head trauma, including shaken baby syndrome, needs a thorough evaluation for a definite diagnosis, which might include social worker intervention and judicial investigation. Thus, the diagnosis might not be promptly established and recorded in the EDs. However, such limitations exist in most database studies on non-accidental injuries^11,22,31^. Furthermore, there was no missing data in the intention and mechanism categories, and data coded as unknown comprised 1.5% and 2.1% of each category. Thus, non-accidental injury based on this definition was considered representative of the study purpose. Second, the PATOS database included only patients in the ED. Patients who had been treated in outpatient departments or other non-ED venues and those who did not avail medical care could not be evaluated. Third, the PATOS database is a multi-country, hospital-based database and not a population surveillance database. Although future population-based research representing the national situation is warranted, the present (first report) pan-Asian study still provides valuable information for pediatric care and public health in the reduction and prevention of non-accidental injury in Asian countries.

## Conclusions

In this pan-Asian study, the background incidence of pediatric non-accidental injuries presenting to the ED was 2.81%, and the places of the incidents and events varied with age and sex. Although the overall mortality rate was lower than that in Western countries, it remained high among preschool children. Abdominal and multiple injuries were associated with major trauma.

## Supplementary Information

Supplementary Information.

## Data Availability

The datasets used and/or analyzed during the current study are available from the corresponding author on reasonable request.

## Abbreviations

ED: Emergency department
EMR-ISS: Excess mortality ratio-adjusted injury severity score
PATOS: Pan-Asian Trauma Outcome Study
