# Supplementary material for: Characteristics of non-accidental injuries in children and adolescents in Asia: a cross-national, multicenter cohort study
Source: Sci Rep. 2023 Apr 23;13:6602. doi: 10.1038/s41598-023-33471-x (PMC10123055; doi:10.1038/s41598-023-33471-x)
Supplement: Supplementary file 1 — Supplementary Information. [file 41598_2023_33471_MOESM1_ESM.pdf]

Characteristics of Non-Accidental Injuries in Children and Adolescents in Asia: a cross-national, multicenter cohort study

Po-Yuan Wang, MD[1], *Wei-Chieh Tseng, MD[2, 3], Meng-Chang Lee, MD[4], Li-Min Hsu, MD[4], Sang Do Shin[5], Sabariah Faizah Jamaluddin[6], Hideharu Tanaka, MD, PhD[7], Do Ngoc Son[8, 9, 10], Ki Jeong Hong[5], Sattha Riyapan[11], Ali Haedar[12], *Wen-Chu Chiang, MD, PhD[3, 13] & the PATOS Clinical Research Network

Affiliations:

[1] Department of Pediatrics, Far Eastern Memorial Hospital, New Taipei City, Taiwan.

[2] Graduate Institute of Clinical Medicine, Medical College, National Taiwan University, Taipei, Taiwan.

[3] Department of Emergency Medicine, National Taiwan University Hospital, Taipei, Taiwan.

[4] Department of Traumatology and Critical Care, National Taiwan University Hospital, Taipei, Taiwan.

[5] Department of Emergency Medicine, Seoul National University College of Medicine and Hospital, Seoul, Korea.

[6] Faculty of Medicine, Universiti Teknologi MARA, Batu Caves, Malaysia.

[7] Graduate School of Emergency Medical Service System, Kokushikan University, Tokyo, Japan.

[8] Center for Critical Care Medicine, Bach Mai Hospital, Hanoi, Vietnam.

[9] Department of Emergency and Critical Care Medicine, Hanoi Medical University, Hanoi, Vietnam.

[10] Faculty of Medicine, University of Medicine and Pharmacy, Vietnam National University, Hanoi, Vietnam.

[11] Department of Emergency Medicine, Faculty of Medicine, Siriraj Hospital, Bangkok, Thailand.

[12] Department of Emergency Medicine, Faculty of Medicine, Universitas Brawijaya, Malang, Indonesia.

[13] Department of Emergency Medicine, National Taiwan University Hospital Yunlin Branch, Douliu City, Taiwan.

Corresponding authors:

Wei-Chieh Tseng, MD, National Taiwan University Hospital, No. 7, Chung-Shan South Road, Taipei,

Taiwan 100. Tel: 886-2-23123456#262648; Fax: 886-2-23223150; E-mail: littlecardiologist@gmail.com

Wen-Chu Chiang, MD, PhD, National Taiwan University Hospital, No. 7, Chung-Shan South Road, Taipei, Taiwan 100. Tel: 886-2-23123456#262831; Fax: 886-2-2322-3150; E-mail: drchiang.tw@gmail.com

*The two authors are both corresponding authors and contributed equally to this work.

Supplementary Table S1. Characteristics of alcohol and psychoactive drug or substance use.

	Preschool (0~6 years)	Children (7~12 years)	Adolescents (13~19 years)
Numbers	23	27	401
Patient			
Alcohol use			
Suspect	1 (4)	1 (4)	85 (21.2)
Confirmed	1 (4)	0 (0)	24 (6.0)
No use	18 (78)	25 (93)	226 (56.4)
Unknown	3 (13)	1 (4)	66 (16.5)
Psychoactive drug/substance use			
Suspect	0 (0)	0 (0)	7 (1.7)
Confirmed	0 (0)	0 (0)	0
No use	19 (83)	26 (96)	319 (79.6)
Unknown	4 (17)	1 (4)	75 (18.7)
Injurer			
Alcohol use			
Suspect	0 (0)	0 (0)	26 (6.5)
Confirmed	0 (0)	0 (0)	3 (0.7)
No use	9 (39)	5 (19)	66 (16.5)
Unknown	14 (61)	22 (81)	306 (76.3)
Psychoactive drug/substance use			
Suspect	0 (0)	0 (0)	1 (0.2)
Confirmed	0 (0)	0 (0)	0 (0)
No use	9 (39)	5 (19)	85 (21.2)
Unknown	14 (61)	22 (81)	315 (78.6)

Supplementary Table S2. Characteristics of Dead Patients.

**Patient**	**1**	**2**	**3**	**4**
Country	Korea	Korea	Korea	Thailand
Age (year)	0	0	15	17
Sex	Male	Male	Male	Female
Prehospital CPR	Yes	Yes	Yes	Yes
Cardiac arrest at triage	No	Yes	No	Yes
CPR at ED	Yes	Yes	Yes	Yes
EMR-ISS	25	16	9	NA
Pre-existing comorbidity	No	No	No	No
Place of injury	Home	Home	Home	Commercial area
Mechanism	Fall	Choking/hanging	Fall	Stab/cut
Location of Injury	Head	Thorax	Head and face	Neck and upper extremity
Type of Injury	Fracture, organ injury	Organ injury	Organ injury	Cut (penetration)
Operation	Yes	Yes	Yes	Yes
Death place	ED	ICU	ED	ED

CPR: cardiopulmonary resuscitation; ED: emergency department; ICU: intensive care unit; NA: not available.

Supplementary Figure S1. Percentage of non-accidental injury events in the population, by age groups

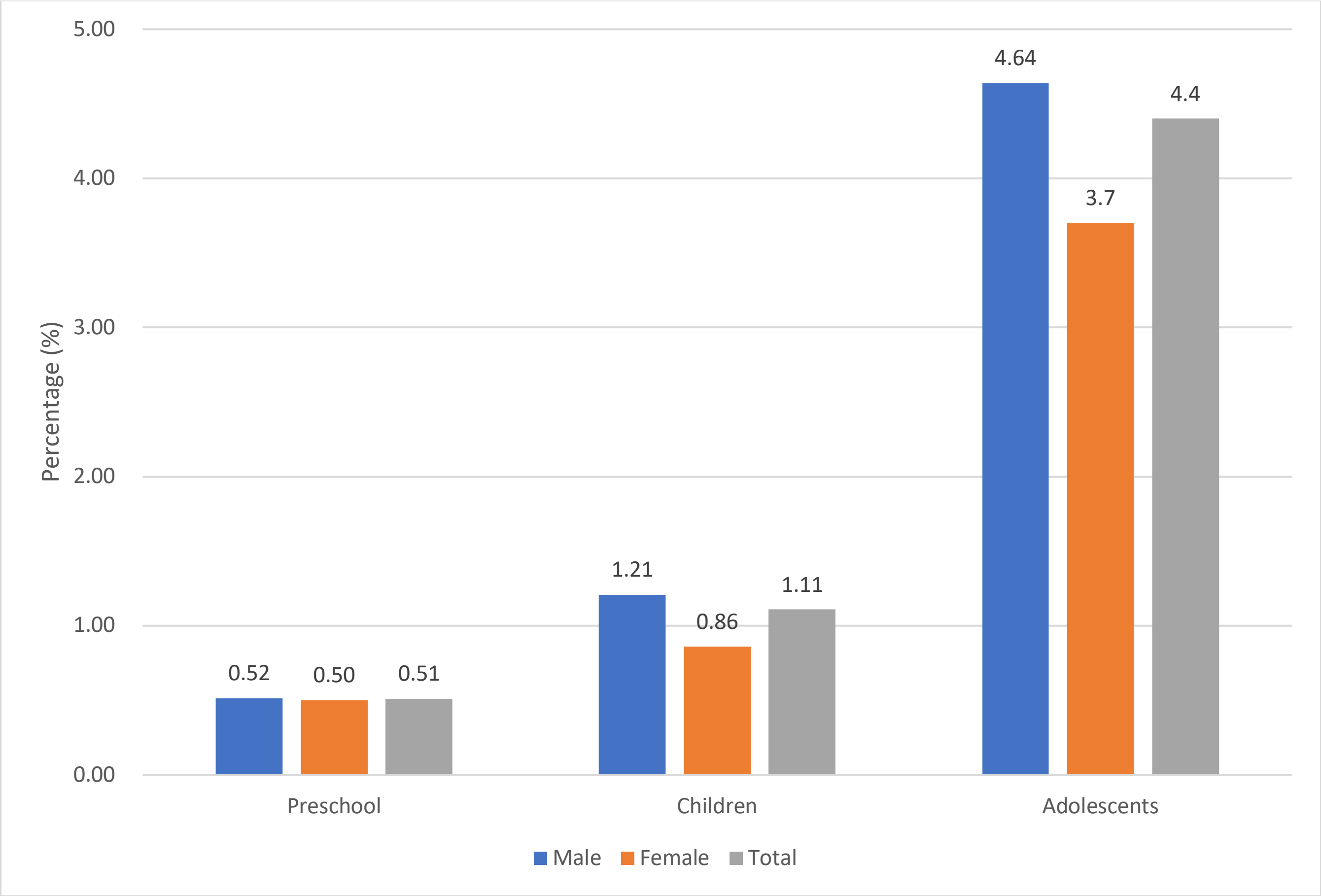

Supplementary Figure S2. Place distribution of non-accidental injury. (a) Total (N = 451), (b) male (n = 347), (c) female (n = 104).

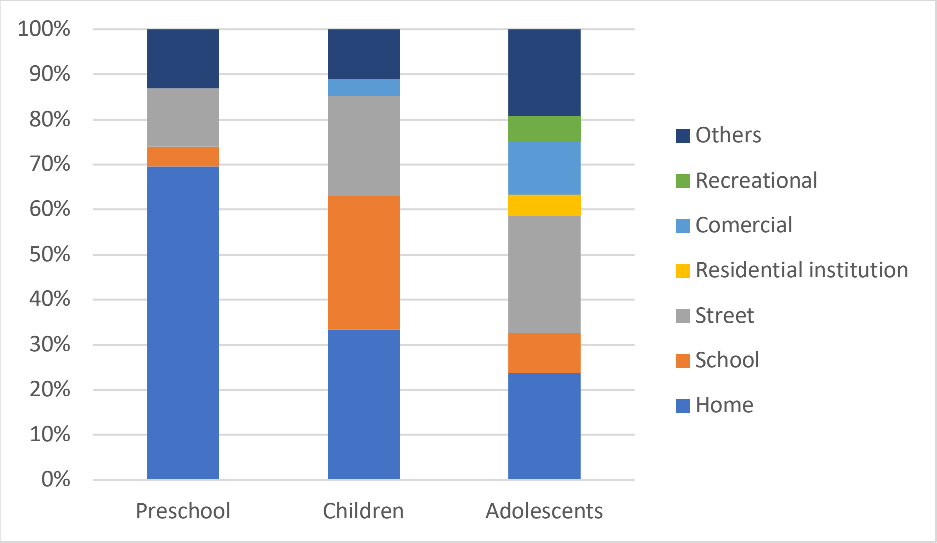

(a)

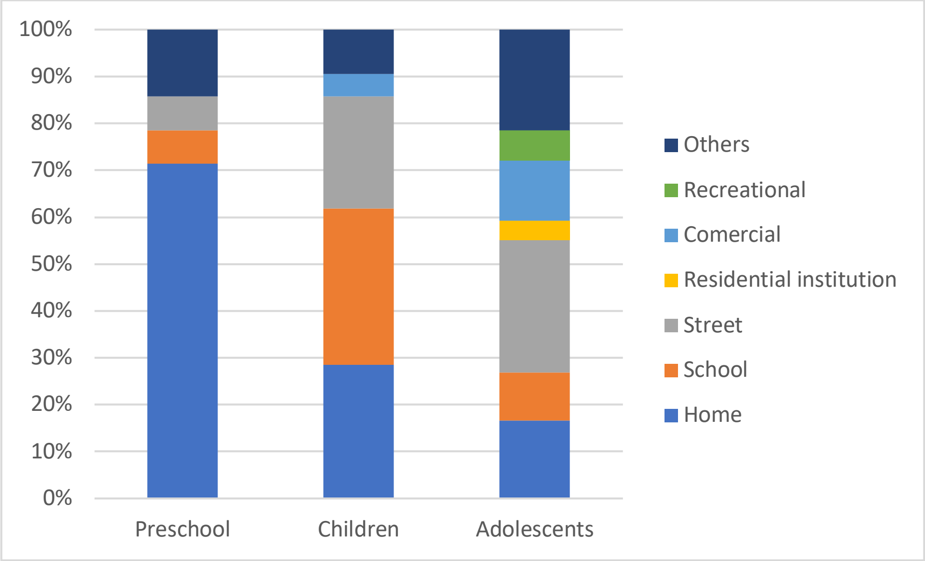

(b)

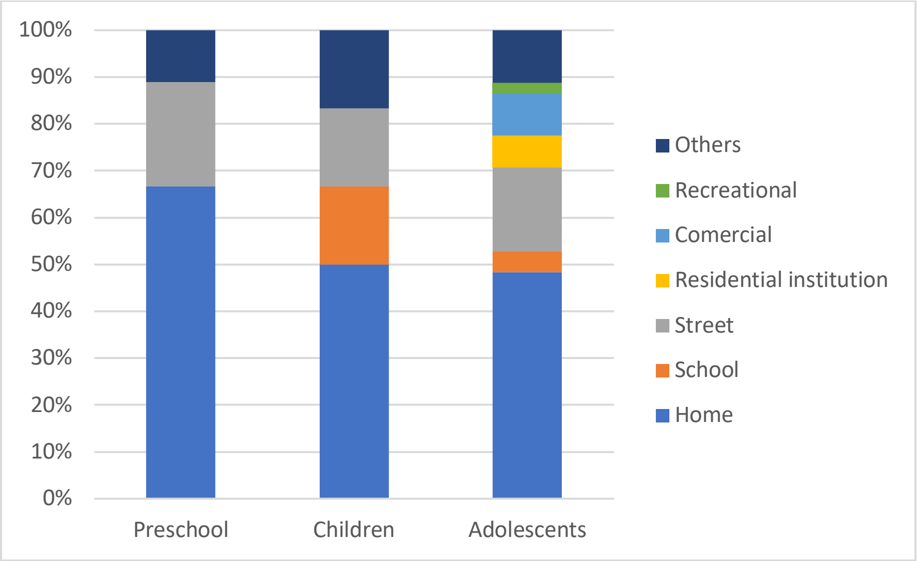

(c)
